# Design, Characterization, and Preparation of New Smart Photoactive Polymers and Their Capacity for Photodynamic Antimicrobial Action in Organic Film

**DOI:** 10.3390/polym17091247

**Published:** 2025-05-03

**Authors:** Oscar G. Marambio, Franco I. Barrera, Rudy Martin-Trasancos, Julio Sánchez, Christian Erick Palavecino, Guadalupe del C. Pizarro

**Affiliations:** 1 Departamento de Química, Facultad de Ciencias Naturales, Matemáticas y Medio Ambiente, Universidad Tecnológica Metropolitana, J.P. Alessandri 1242, Ñuñoa, Santiago 7800002, Chile; 2 Departamento de Química de los Materiales, Facultad De Química y Biología, Universidad de Santiago de Chile, Avenida Libertador Bernardo O’Higgins n 3363, Estación Central, Santiago 9170002, Chile; 3 Departamento de Química Orgánica, Facultad de Química y de Farmacia, Pontificia Universidad Católica de Chile, Santiago 7820436, Chile; 4 Laboratorio de Microbiología Celular, Centro de Ciencias Médicas aplicadas, Facultad de Medicina y Ciencias de la Salud, Universidad Central de Chile, Lord Cochrane 418, Santiago 8330546, Chile

**Keywords:** light-sensitive materials, antimicrobial activity, spiropyrans stimulus-responsive copolymers, morphological surface characteristics

## Abstract

The photosensitive properties of smart photoactive polymers give them a wide range of potential applications across various fields. This study focuses on designing polymeric systems that incorporate hydrophilic polymers, with the primary goal of adapting these materials for biological applications. Specifically, it aims to contribute to the development of photochromic materials for optical processing, utilizing both molecular and macromolecular components. Additionally, this study evaluates the effectiveness of photoactive polymers in photodynamic therapy (PDT). It details the synthesis and characterization of photoactive copolymers derived from maleic anhydride (MAn) combined with vinyl monomers such as 2-methyl-2-butene (MB) and 1-octadecene (OD), as well as the organic compound 1-(2-hydroxyethyl)-3,3-dimethylindoline-6-nitrobenzopyran (SP). The two novel optically active alternating polymeric systems, poly(maleic anhydride-*alt*-octadecene) and poly(maleic anhydride-*alt*-2-methyl-2-butene), were functionalized with SP through an esterification process in a 1:1 monomer feed ratio, using pyridine as a catalyst. This methodology incorporated approximately 100% of the photoactive molecules into the main acrylic chain to prepare the alternating copolymers. These copolymers were characterized by UV-visible, FTIR, and 1H-NMR spectroscopy and analysis of their optical and thermal properties. When exposed to UV light, the photoactive polymer films can develop a deep blue color (566 nm in the absorption spectra). Finally, the study also assesses their capacity for photodynamic antimicrobial action in organic film. Notably, the photoactive P(MAn-*alt*-2MB)-***PS*** significantly enhances the photodynamic antimicrobial activity of the photosensitizer Ru(bpy) against two bacterial strains of *Staphylococcus aureus*, reducing the minimum inhibitory concentration (MIC) from 2 µg/mL to 0.5 µg/mL. Therefore, 4 times less photosensitizer is required when mixed with the photoactive polymer to inhibit the growth of antibiotic-sensitive and -resistant bacteria.

## 1. Introduction

Materials with photochromic properties are recognized as excellent candidates for various technological applications, including optical information storage devices, optical switches, and other materials that exhibit photosensitivity when exposed to light of appropriate frequency and intensity [1]. 

In this regard, the synthesis and copolymerization of photoactive monomers derived from N-(2-methacryloyloxyethyl)-6-nitrospirobenzopyranindoline and methyl methacrylate by atom transfer radical polymerization have been reported [2]. Indeed, some of these compounds have evolved into convenient building blocks for producing commercial ophthalmic lenses, as well as the development of switches and memories, stimulating intense and up-and-coming interdisciplinary research.

Furthermore, a new optically active polymeric methacrylate has been synthesized and investigated, in which the (S)-3-hydroxypyrrolidinyl group, linked through the nitrogen atom to an azopyridine chromophore, was incorporated, in combination with a polymeric methacrylate incorporating the photoisomerizable spiropyran chromophore in the side chain. This system can be modulated in an acidic medium by the protonation of the azopyridine fractions through the photoisomerization of the spiropyran component, allowing it to behave as a chiroptical switch [3].

Similarly, the synthesis of 1′, 3′, 3′-trimethylspiro [2H-1]-benzopyran-2,2′-indoline derivatives with H, Br, I, and NO2 substituents at the 6- and 8-positions of the benzopyran ring has been reported [4]. These compounds were obtained by the condensation of 1,3,3-trimethyl-2-methylindoline with 2-hydroxybenzaldehyde, which was substituted in five positions with several electron-withdrawing groups. Among these, the 6-nitro-spiro benzopyran indoline showed the highest photochromatic activity. It was also established that the absorption of the compound in the visible region of the spectrum, in the excited state, was independent of solvent polarity; however, the extent of stability of the colored form increased with an increase in solvent polarity [4]. On the other hand, the search for a variety of new functional materials, such as films with highly regular patterning, has received significant interest due to their potential use in material technology [5,6] where porous films might find applications in membranes and [7] in photonic [8] and/or optoelectronic devices [9]. Exploring light-responsive molecules in devices typically requires immobilization on a surface through a linker that does not interfere with the structures’ light-switching behavior. This has been achieved for photoswitchable molecules by forming self-assembled monolayers (SAMs) [10] and bilayers [11], as well as by incorporating SAMs into polymer films [12,13] and polymer beads. The reversibility of these macroscopic properties results from photoinduced transformations at the molecular level [14,15]. These processes can be explored to tune optical signals, thereby offering the opportunity to design and implement photonic devices for optical processing based on molecular components [15]. The change in chemical structure allows it to absorb in a specific spectrum region, typically in the visible range, from which it returns to its basal state after a short period under the influence of radiation or a thermal stimulus [16,17]. The terms “positive” and “negative” photochromism are generally used to indicate photoinduced coloration and decoloration processes, respectively. However, both transformations must be reversible by definition [17]. 

On the other hand, maleic anhydride is an organic compound that can react with other monomers to form copolymers; however, it cannot undergo homopolymerization [18]. When it interacts with other vinyl monomers, maleic anhydride produces alternating polymers during copolymerization. This formation of alternating polymers results from a strong donor–acceptor interaction between maleic anhydride and the other monomer, along with maleic anhydride’s low self-interaction reactivity [19].

Spiropyrans (***SP***s), first described by Fischer and Hirshberg in 1962, [20] are one of the most studied classes of photoswitchable compounds. The irradiation of ***SP*** compounds with near-UV light [21] or their electro-oxidation [22] induces the heterolytic cleavage of the spiro carbon–oxygen bond, leading to ring opening and conversion into merocyanine (***MC***). The intense absorption in the visible region of open-form ***MC*** has led to the advanced study of ***SP*** compounds in photochromic [16], molecular optoelectronic [23], and optobioelectronic systems [24], as well as chemical sensing [25].

In this sense, it is essential to highlight the potential application of these materials in biomedicine, due to the progressive accumulation of bacteria with high resistance to antibiotics, which results in progressively fewer antimicrobial alternatives for effective treatment; for this reason, there is a need for materials to complement treatment with antibiotics. In this regard, the emergence of multidrug resistance (MDR) in pathogenic bacteria is one of the most pressing global threats to human health in the 21st century. In the US, almost 23,000 persons die annually due to antibiotic-resistant infections [26]. As bacteria accumulate progressively more resistance factors, this results in progressively fewer antimicrobial alternatives for effective treatment [27]. Hence, the availability of new treatments is indispensable to prevent morbidity and mortality caused by MDR infectious agents. In this regard, photodynamic therapy (PDT) has the advantage of being locally activated using phototherapy devices [28]. PDT utilizes photosensitizer molecules, oxygen, and light to induce non-specific photo-oxidative stress, led by the production of reactive oxygen species (ROS), which inactivate bacteria non-specifically. One bacterial strain that represents a severe threat to public health is the Gram-positive strain *Staphylococcus aureus*. *S. aureus* is one of the most significant MDR bacteria responsible for health-associated infections (HAIs) (30%) [29,30,31,32]. Moreover, MDR strains are responsible for the more prevalent HAIs associated with surgical wound infection, urinary tract infection (UTI), skin wound infections, and pneumonia [33,34,35,36].

This work aims to contribute to the design and implementation of photonic devices for optical processing based on molecular and macromolecular components, and subsequently to evaluate the utility of photoactive polymers in photodynamic therapy (PDT). This work describes the design, synthesis, characterization, and application of two novel photoactive copolymers derived from maleic anhydride (MAn) with vinyl monomers such as 2-methyl-2-butene (MB) and 1-octadecene (OD) and the organic photosensitive compound -(2-hydroxyethyl)-3,3-dimethylindoline-6-nitrobenzopyran (***SP***). Both optically active alternating polymeric systems, poly(maleic anhydride-*alt*-octadecene) and poly(maleic anhydride-*alt*-2-methyl-2-butene), were functionalized with ***SP*** through an esterification reaction. The optically active polymer carries a photoisomerizable spiropyran chromophore in the side chain, linked through the oxygen atom to the polymer chain.

Finally, we focused on determining its ability to inhibit the growth of the MSSA and MDR-MRSA strains of *S. aureus*, the primary cause of superficial infections. This leads to the formation of a solid matrix that, when combined with a photosensitizer, facilitates its use in sanitary pads, hydrogels, or dressings that aid in treating or preventing superficial infections where light can easily activate the photosensitizer. The light-emitting properties of the polymers were characterized by blue and violet colors when exposed to UV light. They were subsequently used in an assay to verify their antimicrobial properties in photodynamic therapy (PDT). These macromolecular components exhibited an evident photon transfer process; based on this behavior, they were investigated with a photosensitizer molecule for PDT. These copolymers were characterized using spectroscopic techniques, such as FT-IR and 1H-NMR, optical microscopy, UV-visible spectroscopy, and thermal analysis techniques, such as TGA.

## 2. Experimental Design

### 2.1. Reagents

Maleic anhydride (MAn), 2-methyl-2-butene (2MB), 1-octadecene (OD), Tetrahydrofuran (THF), benzoyl peroxide (BPO), diethyl ether, magnesium sulfate, sodium carbonate, hydrochloric acid, chloroform, acetone, dimethyl sulfoxide, and methanol were used (Merck, Stuttgart, Darmstadt, Germany). 2,3,3-trimethylindoline, 2-bromoethanol, 2-butanone, trimethylamine, and 2-hydroxy-5-nitrobenzaldehyde *were commercially obtained from Sigma-Aldrich* (St. Louis, MO, USA), *and no further purification was performed before the synthesis of copolymers.*

### 2.2. Characterization 

The absorption spectra of the films were recorded at 25 °C, between 250 and 700 nm, using a Perkin Elmer Lambda 35 spectrophotometer. The FT-IR spectrums were recorded on a Perkin Elmer Spectrum Two spectrometer with a UATR unit coupled in the range of 4000 to 500 cm^−1^, with a resolution of 1 cm^−1^ (Waltham, MA, USA). Photoluminescence (PL) measurements were performed at room temperature by a fluorescence spectrometer system (Perkin Elmer, model 134 LS 55, Waltham, MA, USA). The number average (*M*_n_), weight average (*M*_w_), molecular weights, and polydispersity (*M*_w_/*M*_n_) of the polymers were determined by size exclusion chromatography (SEC) using a Shimadzu LC 20 instrument equipped with an RI detector (Kioto, Japan). An optical microscopy LEICA Model DM2000 LED with an LEICA MFC 170 HD camera was used. The camera was set to an automatic exposure of 500.00 ms, a saturation of 120, and a gamma of 0.00. The image surface was 549.45 μm × 412.09 μm. For image acquisition under fluorescence, the aperture was 1/3 and the focus was 3/3. The films were prepared using a chamber Darwin model PH9-DA with the relative humidity (RH) controlled at 75% and the temperature maintained at 25 °C. 

### 2.3. Procedure for Photochromic Agent Synthesis

**Figure 1:**
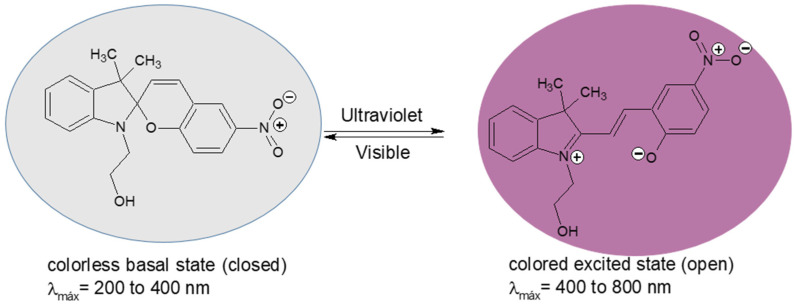
Photoinduced interconversion of 1-(2-hydroxyethyl)-3,3-dimethylindoline-6-nitrobenzopyran by UV-Vis irradiation.

The synthesis of the photochromic agent 1-(2-hydroxyethyl)-3,3-dimethylindoline-6-nitrobenzopyran (SP) was carried out in two steps (a and b), as described below (see Figure 1). Figure S1: (a) Synthesis of 1-(2-hydroxyethyl)-2,3,3-trimethylindolenine bromide and (b) 1-(2-hydroxyethyl)-3,3-dimethylindoline-6-nitrobenzopyran.; Figure S2. (a) FT-IR of 1-(2-hydroxyethyl)-3,3-dimethylindoline-6-nitrobenzopyran, (b) 1H-NMR spectrum of 1-(2-hydroxyethyl)-3,3-dimethylindoline-6-nitrobenzopyran. The synthesis yield was 70.6%, and structural characterization was conducted using FT-IR and 1H-NMR, in accordance with the literature consulted [37].

(a) Synthesis and characterization of 1-(2-hydroxyethyl)-2,3,3-trimethylindolenine bromide salt: The procedure was carried out in a Schlenk tube, to which 4.0 mL (25 mmol) of 2,3,3-trimethylindolenine was added, and then 1.8 mL (25 mmol) of 2-bromoethanol in 3.16 mL (35 mmol) of 2-butanone as a solvent. Subsequently, the mixture was degassed under an inert atmosphere, frozen with liquid nitrogen, and then subjected to several cycles of vacuum and thawing. The synthesis tube was placed in an oil bath at 78 °C with constant stirring for 10 h. Then, at room temperature, the mixture was filtered, yielding a pink solid. This was purified by extraction in a benzene Soxhlet for 24 h until the solution became colorless, yielding 70.6%. The chemical shifts showed the following signals at (δ, ppm, CDCl_3_): 1.65 (9H, singlet, -CH_3_-); 3.15 (1H, singlet, -OH); 4.20 (2H, triplet, CH_2_-OH); 4.89 (2H, triplet, CH_2_-N); and 7.57 (4H, multiplet, aromatic ring). The signals confirmed the molecular structure of 1-(2-hydroxyethyl)-2,3,3-trimethylindolenine bromide.

(b) Synthesis and characterization of 1-(2-hydroxyethyl)-3,3-dimethylindoline-6-nitrobenzopyran (***SP***): In a 250 mL three-neck round-bottom flask equipped with a magnetic stirrer and condenser, 2 g (7.04 mmol) of 1-(2-hydroxyethyl)-2,3,3-trimethylindolenine bromide, 1.2 g (7.04 mmol) of 2-hydroxy-5-nitrobenzaldehyde, and 4 mL of triethylamine (28.16 mmol) in 20 mL of ethanol were added, and heated until boiling (78 °C) for 4 h in an oil bath. After 4 h of reaction, the mixture was allowed to cool, and the ethanol was evaporated using a rotary evaporator.

The product was extracted in a separator funnel with a solution of 10% HCl and chloroform in equal volumes, in order to recover the organic phase from where it was located. After this, the product was dried in the presence of magnesium sulfate, filtered, and the chloroform evaporated, yielding 80.3%. The product obtained was purple crystals.

Moreover, FT-IR exhibited the following vibration band signals υ (cm^−1^, KBr): 1088 (flexion -C-O-C-); 1335 (symmetric stretch Ar-NO_2_); 1510 (asymmetric stretching Ar-NO_2_); 1603 (stretching -C=C-); 1930–1836 (Ar-H aromatic overtones); 2961 (stretching C-H; CH-, -CH_2_-, -CH_3_); 3068 (stretching =C-H); and 3365 (O-H stretch -CH_2_OH). The ^1^H-NMR exhibited the following chemical shifts at (δ, ppm, CDCl_3_): 1.12 [3H, singlet, -CH_3_-]; 1.22 [3H, singlet, -CH_3_-]; 1.53 [1H, singlet, -OH]; 3.46 [2H, multiplet, CH_2_-N]; 3.71 [2H, triplet, CH_2_-OH]; 5.84 [1H, doublet, H (i)]; 6.57 [1H, doublet, H (h)]; 6.71 [1H, doublet, H (g)]; 6.84 [2H, triplet, H (e), H (f)]; 7.01 [1H, doublet, H (d)]; 7.18 [1H, triplet, H (c)]; and 7.97–7.92 [2H, multiplet, H (a) and H (b)]. The signals observed in the spectrum were characteristic of the photochromic compound 1-(2-hydroxyethyl)-3,3-dimethylindoline-6- nitrobenzo pyran (***SP***).

### 2.4. Characterization of **SP** by UV-Visible Spectrophotometry

The colored state of the compound in solution was reached after irradiating the quartz cell with an ultraviolet light for 5 min. A solution of 1 × 10^−5^ M of 1-(2-hydroxyethyl)-3,3-dimethylindoline-6-nitrobenzopyran (***SP***) was prepared, using chloroform as a solvent; 2 mL of this solution was deposited in a quartz cell, and this was placed in the ultraviolet spectrophotometer cell holder, followed by determination of the wavelength values of the compound.

The unimolecular reactions of the organic molecules with photochromic properties involve ring-closing and -opening steps or trans → cis and cis → trans isomerization (see Figure 1). After irradiation with ultraviolet light, their state changes from a colorless basal state (closed) to a colored excited state (open).

### 2.5. Preparation of Photoactive Alternating Copolymers

The preparation of the alternating copolymers P(MAn-*alt*-OD) and P(MAn-*alt*-2MB) was carried out at a 1:1 monomer feed ratio in THF solution. The general procedure for preparing the 1:1 monomer feed ratio was as follows: A 20 mL volume of THF was transferred to a septum-capped, nitrogen-purged flask containing 3.171 g (12.50 mmol) of MAn, 1.196 g (12.50 mmol) of comonomers (OD/2MB), and 0.580 mol-% (140.5 mg) of benzoyl peroxide (BPO). The flask was degassed with freeze cycles and under high vacuum. The solution was placed in a thermoregulated bath at 80 °C for 8 h. The copolymers obtained were then purified by precipitation using a mixture of methanol and ethanol; this procedure was repeated three times, after which the solvents were evaporated from the balloon using a rotary evaporator. The product was dissolved in chloroform (CHCl_3_), and the solution was deposited in a separator funnel with a 10% HCl solution in a 1:1 ratio. This procedure was performed twice. Following the resulting organic phase, a second extraction was performed with chloroform and 10% sodium carbonate (1:1). A soft yellow product was extracted. Subsequently, anhydrous magnesium sulfate was added to the resulting organic phase, and it was left to dry for 30 min to recover the yellow crystals. The yields of the poly(MAn-*alt*-OD) and poly(MAn-*alt*-2MB) copolymers were 70.0% and 80.0%, respectively. As a result, copolymers with molecular weights of 18,200 and 17,300, and polydispersity indices of 1.49 and 1.68, were obtained, respectively. Finally, the dried copolymers were characterized by FT-IR spectroscopy, as well as analysis of their thermal and optical properties. Figure S3: (a) FT-IR spectra of the copolymer P(MAn-*alt*-OD), photochromic agent (***SP***) and P(MAn-*alt*-OD)-***SP***. (b) FT-IR characterization of the copolymers P(MAn-*alt*-2MB) and P(MAn-*alt*-2MB)-***SP***.

Subsequently, the poly(MAn-*alt*-OD) and poly(MAn-*alt*-2MB) copolymers were functionalized with a photochromic compound (***SP***) using a trans-esterification reaction. The experimental procedure was carried in a 100 mL three-neck round-bottom flask equipped with a magnetic stirrer and condenser; 2.0 g (0.036 mmol) of copolymers, 0.013 g (0.036 mmol) of ***SP*** agent, and one drop of H_2_SO_4_ were added to 5 mL of tetrahydrofuran and heated for 4 h. Finally, a purple/yellow-colored product was obtained.

### 2.6. Determination of Antimicrobial Photodynamic Properties 

Photophysical characterization of the P(MAn-*alt*-2MB)-***SP*** copolymer and the Ru(bpy) photosensitizer was performed using a UV-visible spectrophotometer (Shimadzu, UV-1900) and a spectrofluorophotometer (RF-600 Shimadzu). The P(MAn-*alt*-2MB)-***SP*** and Ru(bpy) were suspended in chloroform at 0.2 mg/mL, and a ratio of 8:1 P(MAn-*alt*-2MB)-***SP***:Ru(bpy) was also prepared. Emission measurements were taken by exciting each compound or mixture to their maximum absorption bands.

For PDT, *Staphylococcus aureus* bacteria were cultured in solid or liquid Trypticase soy medium, as required. In a liquid medium, they were grown to an optical density of OD_600nm_ = 0.2–0.4, and for photodynamic therapy (PDT), the bacteria were adjusted to 1 × 10^7^ colony-forming units (CFUs)/mL in a Phosphate-Buffered Saline (PBS) aqueous solution. The bacteria were mixed with the photoactive polymers P(MAn-*alt*-OD)-***SP*** or P(MAn-2MB)-***SP***, with/without photosensitizer Ru(bpy) [38], in various concentrations. Excitation was performed in a light box with 17 mW/cm^2^ for 10 min with a blue LED lamp (450–460 nm) equivalent to 61.2 J/cm^2^. After excitation, bacterial viability was determined by broth microdilution and colony counting on plates after 16–20 h of incubation in Muller–Hinton medium. Bacterial viability was expressed as the mean ± SD in CFU/mL. The MIC of the photosensitizer was determined by mixing 1 × 10^7^ CFU/mL of bacteria with increasing concentrations of the photosensitizer Ru(bpy), between 0.125 and 4 μg/mL.

## 3. Results and Discussion

**Figure 2:**
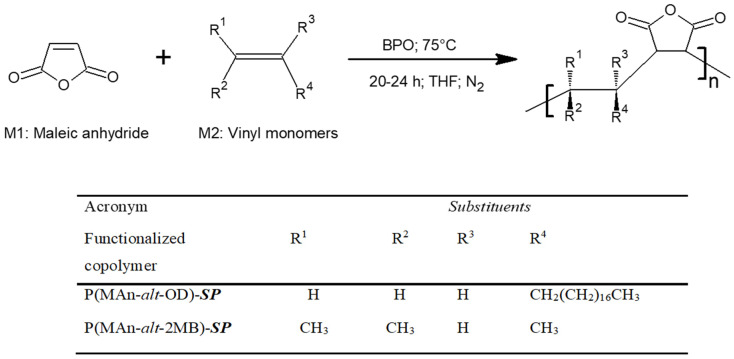
General reaction for synthesis of copolymers. Name and acronyms used for copolymers functionalized with maleic anhydride (MAn).

**Figure 3:**
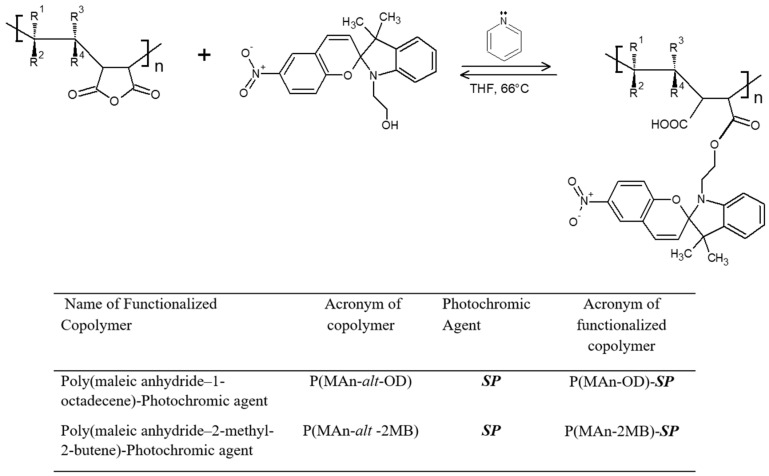
General reaction of copolymer functionalization. Name and acronyms used for copolymers functionalized with maleic anhydride (MAn).

Photoactive copolymers were obtained that contained an ***SP*** moiety and had photoluminescence properties. The copolymers were synthesized in an approximate molar ratio of 1:1, as shown in Figure 2, and their functionalized copolymers are displayed in Figure 3.

### 3.1. Characterization of Alternating Copolymers

#### 3.1.1. Characterization by FT-IR and ^1^H-NMR

The FT-IR spectrum for P(MAn-*alt*-OD) showed the following vibration bands: 2957.5 (stretching =C-H); 2855.0 (stretching C-H; CH-, -CH_2_-, -CH_3_); 1780.0–1720.0 (symmetric stretching >C=O; -CO-O-CO- MAn); 1707.5 (stretching C=O; -COOH); and 1467.0 (bending C-H; -CH_2_-, CH_3_). The FT-IR spectrum of ***SP*** showed the following vibration bands υ (cm^−1^, KBr): 3386.0 (O-H stretch; CH_2_OH); 2960.0 (stretching =C-H); 2860.0 (stretching C-H; CH-, -CH_2_-, -CH_3_); 1780.0–1720.0 (Ar-H aromatic overtones); 1612.5 (stretching -C=C- Ar); 1485.0 (asymmetric stretching N-O; -N=O); and 812.5 and 747.5 (C-H stretching; polysubstituted H-Ar). Meanwhile, the FT-IR spectrum of the P(MAn-*alt*-OD)-***SP*** functionalized copolymer exhibited characteristic absorption bands at 2957.5; 2855.0 (stretching C-H; CH-, -CH_2_-, -CH_3_); 1780.0 (stretching >C=O asymmetric; -CO-O-CO- MAn); 1720.0 (stretch C=O; -COOR ester); 1612.5 (stretch C=C; Ar); 1485.0 (N-O stretch; -N=O); 1467.0 (C-H bending; -CH_2_-, CH_3_); and 812.5 and 747.5 (C-H stretching; polysubstituted H-Ar). These signals are characteristic of the photochromic compound ***SP***, confirming its incorporation in P(MAn-*alt*-OD)-***SP***.

The FT-IR spectrum for P(MAn-*alt*-2MB) exhibited the following vibration bands: 2985.0–2855.0 (stretching C-H; CH-, -CH_2_-, -CH_3_); 1857.5 (stretching >C=O asymmetric; -CO-O-CO- MAn); 1780.0 (stretching >C=O symmetric; -CO-O-CO- MAn); 1719.0 (stretching C=O; -COOH); and 1480.0 and 1390.0 (bending C-H; -CH_2_-, CH_3_).

The FT-IR spectrum of ***SP*** showed the following vibration bands: 3382.0 (tension O-H; -OH from ***SP***); 2985.0–2855.0 (stretching C-H; CH-, -CH_2_-, -CH_3_); 1612.5 (stretching C=C; Ar); 1485.0 (stretching N-O; -N=O); and 807.5 and 750.0 (stretching C-H; H-Ar, polysubstituted). Meanwhile, the FT-IR spectrum of P(MAn-*alt*-2MB)-***SP*** exhibited characteristic absorption bands at 2985.0–2855.0 (stretching C-H; CH-, -CH_2_-, -CH_3_); 1857.5 (stretching >C=O symmetrical; -CO-O-CO- MAn); 1780 (stretching >C=O asymmetric; -CO-O-CO- MAn); 1728.0 (stretching C=O; -COOR ester); 1612.5 (stretching C=C; Ar); 1485.0 (stretching N-O; -N=O); and 807.5 and 750.0 (stretching C-H; H-Ar, polysubstituted). Table 1 summarizes the main vibration bands in the FT-IR spectra for the functionalized copolymers.

The asymmetric and symmetric tension bands for the MAn comonomer ring appeared at 1857.0 cm^−1^ and 1780.0 cm^−1^, evidencing that a fraction of the MAn ring remained in the functionalized copolymer. Meanwhile, functionalization with the photochromic agent was evidenced by the tension bands of the carbonyl (C=O) of the ester at 1720.0 cm^−1^ and 1728.0 cm^−1^ for the copolymers P(MAn-*alt*-OD)-***SP*** and P(MAn-*alt*-2MB)-***SP***, respectively, in addition to the N-O (–NO_2_) bond strain bands at 1460.0 cm^−1^. The carboxylic acid carbonyl tension bands present in both copolymers were not present in the functionalized copolymers.

#### 3.1.2. Characterization by ^1^H-NMR of Copolymers P(MAn-*alt*-OD)-***SP*** and P(MAn-*alt*-OD)-***SP***

**Figure 4:**
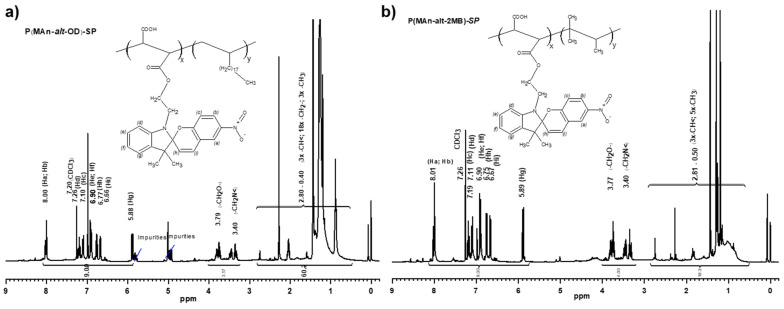
**(a**) NMR^1^H- spectrum of P(MAn-*alt*-OD)-***SP*** copolymer. (**b**) NMR ^1^H-spectrum of P(MAn-*alt*-2MB)-***SP*** copolymer.

The ^1^H-NMR spectrum (δ ppm, CHCl_3_) of the P(MAn-*alt*-OD)-***SP*** copolymer showed the following signals (see Figure 4a): 8.00 (2H, m, Ha and Hb); 7.20(1H, t, Hd); 7.10(1H, d, Hc); 6.90(2H, m, He and Hf); 6.77(1H, d, Hh); 6.66(1H, d, Hi); 5.88(1H, d, Hg); 3.79(2H, m, -CH_2_O-); 3.40(2H, m, -CH_2_N<); and 2.80 to 0.4 (48H, m, 3x –CH<, 18x –CH_2_- and 3x –CH_3_). The characterization of P(MAn- alt-OD) showed the following signals at δ (ppm, DMSO-d6): 2.2 to 1.7 (2H, m width, MAn), and 1.6 and 0.6 (40H, S wide, >CH-, -CH_2_-, -CH_3_). The ^1^H-NMR spectrum (δ ppm, CHCl_3_) of P(MAn-*alt*- 2MB)-***SP*** showed the following signals: 8.01(2H, m, Ha, Hb); 7.19(1H, t, Hd); 7.11(1H, d, Hc); 6.90(2H, m, He, Hf); 6.75(1H, d, Hh); 6.67(1H, d, Hi); 5.89(1H, d, Hg); 3.77(2H, m, -CH_2_O-); 3.40(2H, m, -CH_2_N<); and 2.80 to 0.5 (18H, m, 3x –CH<, 5x –CH_3_) (see Figure 4b).

#### 3.1.3. Degrees of Functionalization

The degree of functionalization of the two copolymers was determined by designing systems of equations relating the quantities of aliphatic protons, methylene protons, and/or aromatic protons of the copolymer matrices with their integrals and their chemical shifts in ^1^H NMR spectroscopy. Equations (1)–(4) were established for this purpose.

(1) $$W_{ar}=\frac{I_{aliph}*n_{4}-I_{ar}*n_{1}}{I_{ar}*n_{2}}$$

(2) $$W_{meth}=\frac{I_{aliph}*n_{3}-I_{meth}*n_{1}}{I_{meth}*n_{2}}$$

(3) $$y=\frac{W}{1+W}$$

(4) x+y=1

where

- $n_{1}$ = the number of aliphatic protons in fraction X;
- $n_{2}$ = the number of aliphatic protons in fraction Y;
- $n_{3}$ = the number of methylene protons;
- $n_{4}$ = the number of aromatic protons;
- $I_{aliph}$ = integral aliphatic protons;
- $I_{ar}$ = integral aromatic protons;
- $I_{meth}$ = integral methylene protons;
- $W_{ar}$ = a numerical value, the mathematical operation of η_1_, η_2_, η_4_, $I_{aliph}$, $I_{ar}$;
- $W_{meth}$ = a numerical value, the mathematical operation of η_1_, η_2_, η_3_, $I_{aliph}$, $I_{meth}$;
- x = the comonomer fraction in X;
- y = the comonomer fraction in Y.

#### 3.1.4. Functionalization of P(MAn-*alt*-OD)-***SP*** and P(MAn-*alt*-2MB)-***SP***

To establish the copolymer composition of the functionalized copolymers, the integrals of each aliphatic fraction of the copolymers were considered versus the fractions of the numerical values $W_{meth}$ and $W_{ar}$, and the degree of functionalization was determined. As a result, similar values for X and Y were obtained by both methods (see Table 2). The results obtained for fractions X and Y showed that the functionalization of the MAn comonomer (fraction X) was 40% and 49%, respectively.

#### 3.1.5. Characterization of Copolymers by UV-Visible Spectrophotometry

**Figure 5:**
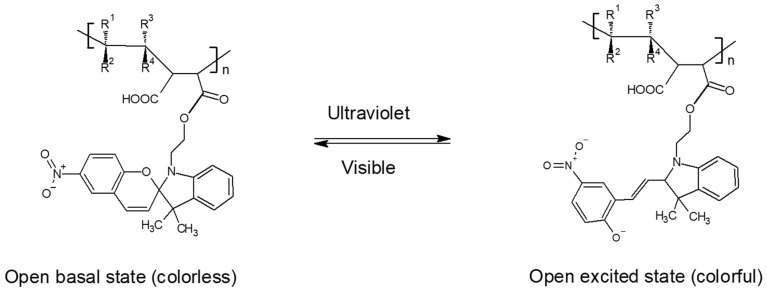
Photochromism reaction of functionalized copolymers.

Figure 5 shows the photochromic equilibria of the functionalized copolymers; when irradiated by ultraviolet light, they changed from a colorless basal state (closed) to a colorful excited state (open).

**Figure 6:**
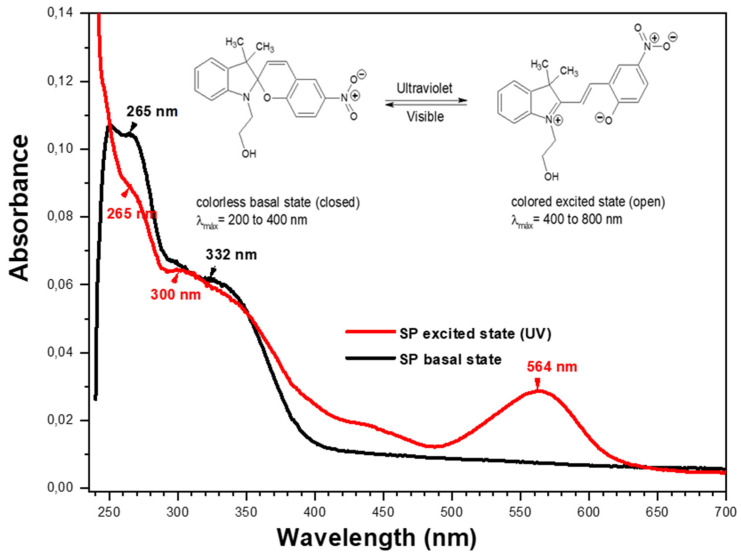
Absorption changes of photoactive agent (3 mg·mL^−1^, in THF), before irradiation (black line) and after irradiation at 365 nm (red line).

In Figure 6, it is possible to observe the isomeric equilibrium of the photochromism of ***SP***. The basal UV spectrum of ***SP*** presents two λmax, at 265 nm and 332 nm, and the spectrum in its excited state presents three λmax, at 265 nm, 300 nm, and 564 nm. These data demonstrate photochromic isomeric balance, since the λmax at 265 nm and 300 nm are maintained, and a new λmax is formed at 564 nm, visibly evidenced by blue coloration. 

**Figure 7:**
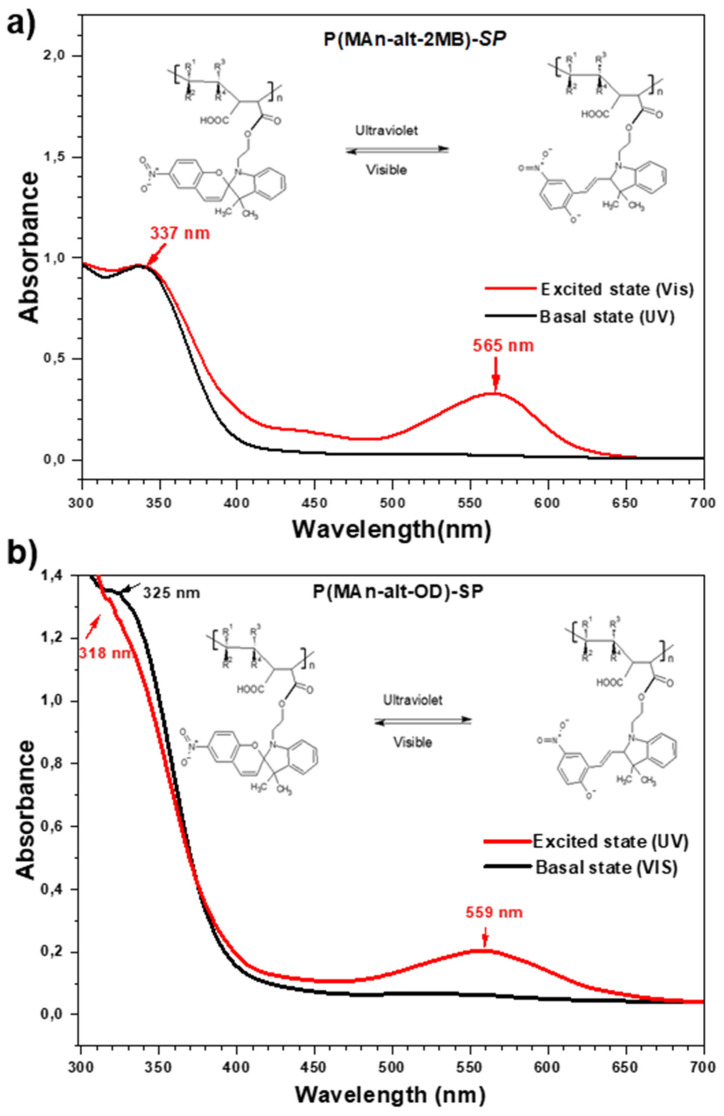
Absorption changes in photoactive-polymer solution (3 g/L in THF) (**a**) before irradiation and (**b**) after irradiation at 365 nm.

On the other hand, the solution of photoactive copolymers showed photoinduced reversible interconversion upon UV-Vis irradiation. As a result, the absorbance of the photochromic solution could be modulated simply by turning an ultraviolet source on and off. The spectrophotometry of the copolymers functionalized with ***SP*** generated two isomeric structures in equilibrium. These were formed when the sample in solution was irradiated with ultraviolet light at 365 nm, moving from a colorless basal state (closed molecular state) to a colorful (blue) excited state (open molecular state) (see Figure 7).

Figure 7 shows the effect of irradiation of the functional polymers P(MAn-*alt*-2MB)-***SP*** and P(MAn-*alt*-OD)-***SP*** on the absorption spectrum. After irradiation, the photoinduced formation of colored-state “merocyanine” (***MC***) was responsible for the significant increases in absorbance in the visible region at 560 and 566 nm, respectively, indicating photoconversion from ***SP*** to ***MC*** (open conformation) [39,40]. Table 3 summarizes the λmax in the basal-state and excited-state UV-VIS spectra of the photochromic agent and the functionalized copolymers. 

The basal-state spectrum of the functionalized copolymer P(MAn-*alt*-OD)-***SP*** presented a λmax at 320 nm, and the excited-state spectrum presented two λmax, one at 318 nm and a new signal at 560 nm. These data demonstrate the photochromic isomeric equilibrium of the copolymer, which was evidenced by visible blue coloration. Moreover, the basal-state spectrum of the functionalized copolymer P(MAn-*alt*-2MB)-***SP*** presented three λmax, at 268 nm, 300 nm, and 337 nm, whereas the excited-state spectrum presented four λmax, at 268 nm, 300 nm, 337 nm, and 566 nm (a new signal). Therefore, the photochromic balance of the ***SP*** agent was maintained in the functionalized copolymer. The λmax close to 560 nm showed the breaking of the spiro bond (N-C-O-C) coming from the benzopyran of the photochromic agent and the functionalized copolymers, forming an isomeric structure called merocyanin (MC), which has conjugated π bond systems (conjugated polyenes), with a great resonance effect induced by a nitro group capable of absorbing energy and being excited in the visible light region. This is due to the decrease in the energy through the electronic transition of the double bond system from π to π*, where the excited state has more polarity than the basal state, and radiation is mostly effective, showing a large bathochromic shift and a hypochromic effect of lower intensity. Furthermore, the electronic transition of π to π*, from a basal singlet state to an excited singlet state, is very likely to occur, since the spin of the excited electron is unpaired with its antiparallel spin, and its relaxation mechanism is fast and reversible (see Figure 7). Meanwhile, the electronic excitation of π to π* at the triplet level requires a “forbidden” spin transition to be carried out, which implies that it is unlikely to form, since the spin of the excited electron is unpaired with its parallel spins, and its relaxation mechanism is slow and not easily reversible.

PDT requires light irradiation of a specific wavelength to excite the ***PS*** moiety. As is exhibited in Figure 7, upon irradiation, the ***PS*** moiety in its lowest energy level (ground singlet state, π) is changed to the short-lived excited singlet state (1π*), which can be converted to the long-lived excited triplet state, (3π*). In the presence of ambient oxygen, the triplet state can undergo two types of reaction mechanisms: (I) the transfer of electrons to form toxic reactive oxygen species (such as peroxide, H_2_O_2_, and hydroxyl radicals, etc.); (II) a mechanism involving energy transfer to ground-state triplet oxygen to produce highly reactive singlet oxygen (1O_2_^●^). The two reactions can take place simultaneously. However, not all highly conjugated unsaturated organic molecules can undergo intersystem crossing to produce the triplet state necessary for photochemical reactions to occur [41].

The optical band gap *E*_g_ of the photoactive copolymers was estimated from UV-Vis measurements before and after irradiation at a wavelength of 365 nm, using the Tauc plot equation: (*αhν*) = *A* (*hν − E*_g_)^m^, where *α* is the absorption coefficient, *h* is Planck’s constant, *ν* is the frequency of light, *A* is a constant, *m* is a constant related to the type of optical transition, and E_g_ is the band gap. The absorption coefficient was estimated from *α* = 2.303 *A*/*d*, where *A* is the absorbance and *d* is the compactness of the film [42,43]. The value of *m* = 1/2 was used in the calculations, because direct optical transition was considered for all compounds [44]; therefore, (*αhν*)^2^ versus *hν,* the photon energy, was plotted, and the linear part of the figure was extrapolated by a straight line to (*αhν*)^2^ = 0 to estimate the band gap. Table 4 shows both of the photoactive copolymers’ band gap (*E*_g_) values, estimated before and after irradiation. The band gap value for P(MAn-*alt*-2MB)-***SP*** was higher than for P(MAn-*alt*-OD)-***SP***. After irradiation, the band gap value for P(MAn-*alt*-2MB)-***SP*** decreased at 3.78 eV. The same behavior was observed for P(MAn-*alt*-OD)-***SP***. After irradiation, the band gap values for both functionalized copolymers decreased. During irradiation, the photons reached an intermediate energy level, which expanded the light absorption range into the visible region by generating new surface energy levels in the material. This contributed to the transfer of energy during the activation process, promoting the generation of free electrons, which negatively affect bacteria. This was directly related to the application of photodynamic therapy. In the case of P(MAn-*alt*-2MB), the band gap decreased significantly when the material was exposed to UV-Vis light. The reduction in the band gap could have been due to the development of a new transitional level beneath the conduction band [42].

### 3.2. Thermal Decomposition Analysis by TGA

**Figure 8:**
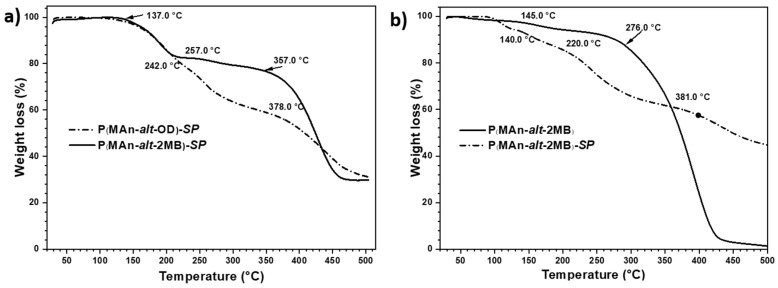
Thermograms of the copolymers and photoactive copolymers (**a**) P(MAn-*alt*-OD) and P(MAn-*alt*-OD)-***SP***, and (**b**) P(MAn-*alt*-2MB) and P(MAn-*alt*-2MB)-***SP***.

The results of the thermal behavior, determined by TGA, of both copolymers and their functionalized derivatives are presented in Figure 8, expressed in percentage mass losses (%) as a function of the increase in temperature. Additionally, the extrapolated thermal decomposition temperature (TDTe) is reported. The thermograms of the copolymers exhibited higher thermal stability than those of the copolymers functionalized with the photochromic agent. The copolymers exhibited two TDTes; the first temperature is attributed to the decomposition of the hydrocarbon chain, and the second TDT is attributed to the decomposition of the maleic anhydride ring. On the other hand, the functionalized polymer P(MAn-*alt*-OD)-***SP*** exhibited two TDTes (137.0 °C and 357.0 °C); the first decomposition temperature is attributed to the bond of the ester group, which is less stable than the other covalent bonds of the macromolecule, and breaks at temperatures above 100 °C. Consequently, this bond was the first to break in the SP-functionalized compound, releasing the SP agent. Subsequently, the second step, at 357.0 °C, involved degradation of the macromolecule’s main chain, decomposition of the hydrocarbon chain of the copolymer, evidenced by a 75% loss of mass (seeFigure 8a). Furthermore, the thermogram of P(MAn-*alt*-2MB) exhibited two TDTes (145.0 °C and 276.0 °C), and the second TDT exhibited total decomposition of the copolymer, with a mass loss reaching 99%. At the same time, the photoactive P(MAn-*alt*-2MB)-***SP*** exhibited three TDTes (140.0 °C, 220.0 °C, and 381.0 °C); the two first are attributed to the decomposition of the ester group and hydrocarbon chain of the copolymer, respectively, and the third, at 381.0 °C, demonstrates greater stability of the maleic anhydride ring with the photochromic agent, evidenced by a 55% loss of mass (see Figure 8b).

### 3.3. Characterization by Fluorescence Optical Microscopy 

**Figure 9:**
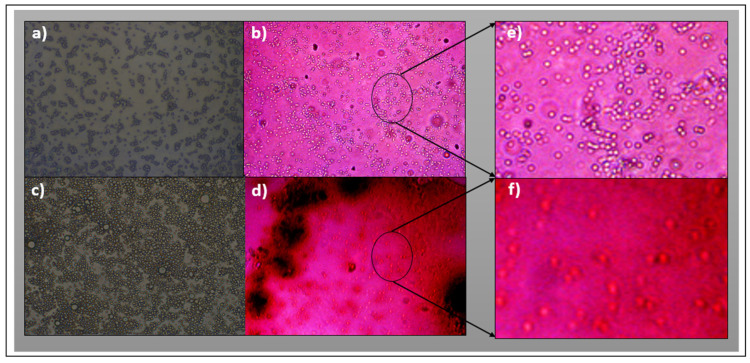
Optical microscope images (OMIs) at 40× magnification in CS2. (**a**) Before and (**b**) after irradiation of P(MAn-*alt*-OD)-***SP*** film; and (**c**) before and (**d**) after irradiation of P(MAn-*alt*-2MB)-***SP*** film. Enlarged images (inset zoom) (**e**,**f**).

Figure 9 shows the surface of the P(MAn-*alt*-OD)-***SP*** and P(MAn-*alt*-2MB)-***SP*** films before and after UV irradiation at 3 g·L^−1^ (in CS_2_), respectively. When exposed to visible light, the images exhibit structured surfaces and show red luminescence. After irradiation, the photoinduced formation of colored-state “merocyanine” (***MC***) is observed, due to incorporation of the ***SP*** moiety into the polymer chain. The optical images of the surface films show a specific pore array on the surface. The ***SP*** moieties grafted to the chain polymer are expected to lead to different surface organization, with varying distribution over the whole surface (Figure 9a–d).

### 3.4. Determination of Antimicrobial Properties Based on Photodynamic Therapy (PDT)

**Figure 10:**
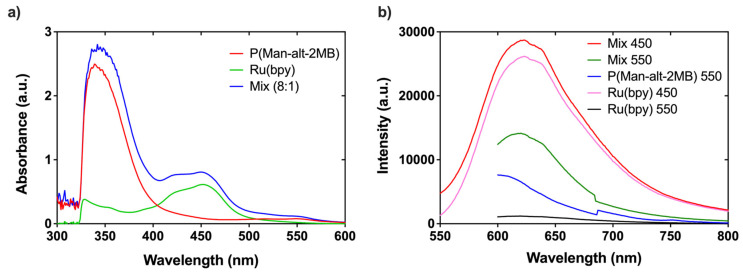
The photophysical properties of P(MAn-*alt*-2MB)-***SP*** and the photosensitizer Ru(bpy). The absorption spectrum (**a**) and the emission spectrum (**b**) are shown.

We characterized the photophysical properties of the polymer composite P(MAn-*alt*-2MB)-*PS* and the photosensitizer Ru(bpy). As shown in the emission spectra in Figure 10a, P(MAn-*alt*-2MB)-***SP*** exhibits absorption maxima at 350 and 550 nm. The photosensitizer displays a single absorption maximum at 450 nm, while the 8:1 Mix of P(MAn-*alt*-2MB)-***SP*** with Ru(bpy) reveals a combination of absorption bands at 350, 450, and 550 nm. This 8:1 ratio was selected because it demonstrated the best performance in the subsequent photodynamics results. As indicated in Figure 10b, the emission spectra of P(MAn-*alt*-2MB)-***SP*** display a maximum emission band at 600 nm when excited at 550 nm. In contrast, Ru(bpy) shows no emission bands when excited at 550 nm. However, the 8:1 ratio exhibits an emission shift towards 620 nm when excited at 550 nm. A significant increase in the intensity band at 620 nm occurs when excited at 450 nm. The intensity of this band is comparable to that observed when Ru(bpy) is excited at 450 nm, its maximum absorption band. During irradiation, the photons reach an intermediate energy level, and a second band forms in the visible region as new surface energy levels are created within the material. This contributes to narrowing of the band gap and facilitates the generation of free radicals during the activation process, promoting energy transfer from triplet oxygen to the singlet state to produce highly reactive singlet oxygen (1O₂^●^), which negatively affects the bacteria. This is directly related to the application of photodynamic therapy. 

**Figure 11:**
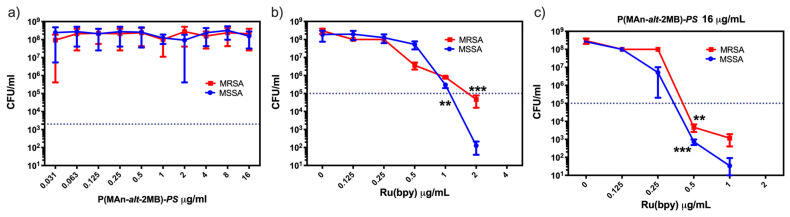
The antimicrobial photodynamic properties of P(MAn-*alt*-2MB)-***SP*** and the photosensitizer Ru(bpy). The photodynamic antimicrobial activity against methicillin-susceptible (RMSSA) and methicillin-resistant (MRSA) *Staphylococcus aureus* was assessed: the intrinsic antibacterial photodynamic activity of P(MAn-*alt*-2MB)-***SP*** was evaluated between 0.031 and 16 μg/mL (**a**), the MIC of the photosensitizer Ru(bpy) was determined between 0 and 4 μg/mL (**b**), and the reduction in MIC for Ru(bpy) was evaluated by mixing it with 16 µg/mL of P(MAn-*alt*-2MB)-***SP*** (**c**). Bacterial viability is expressed as the log_10_ of the mean ± SD, with ** *p* < 0.01 and *** *p* < 0.001 from a two-tailed *t*-test with comparison to control bacteria in the dark.

Since the optically active polymers, P(MAn-*alt*-OD)-***SP*** and P(MAn-*alt*-2MB)-***SP***, present macromolecular components with an evident photon transfer process, their utility for antimicrobial photodynamic therapy (aPDT) was investigated. The PDT technique employed the photoactive copolymer (***PS***) in the presence of the photosensitizer, Ru(bpy), followed by light irradiation with a specific wavelength that could excite the photosensitizer and ***SP*** moiety to cause the production of cytotoxic ROS in the presence of ambient molecular oxygen. The ROS produced during the application of irradiation can exert lethal effects on microbial pathogens. As seen in Figure 11a, the P(MAn-*alt*-2MB)-***SP*** compound showed no intrinsic aPDT activity against MSSA or MRSA *S. aureus* strains. As expected, the photosensitizer Ru(bpy) presented aPDT activity, and the minimum inhibitory concentration (MIC) for inhibiting the MSSA and MRSA *S. aureus* strains was determined to be 2 μg/mL (Figure 11b). The MIC of Ru(bpy) was significantly (*p* < 0.01) improved when mixed with the polymer P(MAn-*alt*-2MB)-***SP*** (Figure 11c), decreasing the MIC to 0.5 μg/mL. This means that 4 times less photosensitizer was required, when mixed with P(MAn-*alt*-2MB)-***SP***, to inhibit the growth of both antibiotic-sensitive and -resistant bacteria. On the other hand, P(MAn-*alt*-OD)-***SP*** showed no significant improvement in the photodynamic activity of the Ru(bpy) photosensitizer. Therefore, because the polymer P(MAn-*alt*-2MB)-***SP*** significantly enhances the aPDT of the Ru(bpy) photosensitizer, it is an excellent complement to treat or prevent infections.

## 4. Conclusions

The results obtained confirm that the preparation of two new alternating copolymers, P(MAn-*alt*-OD) and P(MAn-*alt*-2MB), as well as their photoactive polymers P(MAn-*alt*-OD)-***SP*** and P(MAn-*alt*-2MB)-***SP***, was achieved through free radical polymerization utilizing maleic anhydride with octadecene and 2-methyl-2-butene comonomers. 

Moreover, as part of the photochemical evaluation, the results demonstrate that the photoactive properties of the photochromic compound are slightly altered when incorporated into the polymer chain. The optical and morphological properties of the photoactive copolymers and their relationship with each other were analyzed, demonstrating that the compounds exhibit an evident photon transfer process, characterized by a change in color after irradiation. For both ***SP***-functionalized copolymers, the band gap decreased significantly after UV irradiation. During irradiation, the photons reached an intermediate energy level, which expanded the light absorption range into the visible region by generating new surface energy levels in the material. This contributed to a reduction in the band gap width and facilitated the production of free radicals during the activation process, promoting the generation of free electrons, which negatively affect bacteria. This is directly related to the application of photodynamic therapy. On the other hand, the film copolymers also exhibited light-sensitive behavior under UV light irradiation, showing changes in their optical and morphological properties. The photoactive polymer P(MAn-*alt*-2MB)-***PS*** significantly enhanced the photodynamic antimicrobial activity of the photosensitizer Ru(bpy) against bacterial strains, decreasing the minimum inhibitory concentration (MIC) from 2 μg/mL to 0.5 μg/mL. Therefore, because the polymer P(MAn-*alt*-2MB)-***SP*** significantly enhances the aPDT of the Ru(bpy) photosensitizer, it is an excellent complement to treat or prevent infections. The findings confirm that these materials are promising for next-generation antimicrobial coatings and light-responsive smart materials. 

## Figures and Tables

**Table 1:** The main vibration bands of the functionalized copolymers.

	Maleic Anhydride (MAn)		COOH	Ester -COOR	-NO_2_
Copolymer	Asymmetric (C=O)	Symmetrical (C=O)	Tension (C=O)	Tension (C=O)	Tension (N-O)
P(MAn-*alt*-OD)	---	1780.0	1707.5	---	---
P(MAn-*alt*-OD)-***SP***	---	1780.0	---	1720.0	1460.0
P(MAn-*alt*-2MB)	1857.0	1780.0	1719.0	---	---
P(MAn-*alt*-2MB)-***SP***	1857.0	1780.0	---	1728.0	1460.0

**Table 2:** Degrees of functionalization of P(MAn-*alt*-OD)-***SP*** and P(MAn-*alt*-OD)-***SP.***

Name	n_1_	n_2_	n_3_	n_4_	I_aliph_	I_meth_		I_ar_	X	Y	
P(MAn-*alt*-OD)-***SP***	8.00	10.0	4.00	9.00	48.0	6.06		---	0.40	0.60	
P(MAn-*alt*-OD)-***SP***			8.00	10.0	4.00	9.00	48.0	---	2.53	0.40	0.60
P(MAn-*alt*-2MB)-***SP***	8.00	10.0	4.00	9.00	18.3	4.00		---	0.49	0.51	
P(MAn-*alt*-2MB)-***SP***			8.00	10.0	4.00	9.00	18.3	---	9.00	0.49	0.51

**Table 3:** Maximum wavelength (λmax) of photochromic agent and functionalized copolymers.

Compounds	λmax in Basal State (nm)			λmax in Excited State (nm)			
Photochromic agent	265	332	--	265	300	--	564
P(MAn-*alt*-OD)-***SP***	320	--	--	318	--	--	560
P(MAn-*alt*-2MB)-***SP***	268	300	337	268	300	337	566

**Table 4:** Optical band gap (*E*_g_) *.

Case	*E*_g_ (eV)	
	Before Irradiation	After Irradiation
Photochromic agent (***SP***)	3.00	2.00
P(MAn-*alt*-OD)-***SP***	3.73	3.02
P(MAn-*alt*-2MB)-***SP***	4.53	3.78

* The *E*_g_ was estimated from the Tauc plot equation [43], before and after irradiation at 365 nm.

## Data Availability

Data are contained within the article.

## Supplementary Materials

The following supporting information can be downloaded at: https://www.mdpi.com/article/10.3390/polym17091247/s1, Figure S1: (a) Synthesis of 1-(2-hydroxyethyl)-2,3,3-trimethylindolenine bromide and (b) 1-(2-hydroxyethyl)-3,3-dimethylindoline-6-nitrobenzopyran; Figure S2. (a) FT-IR of 1-(2-hydroxyethyl)-3,3-dimethylindoline-6-nitrobenzopyran, (b) 1H-NMR spectrum of 1-(2-hydroxyethyl)-3,3-dimethylindoline-6-nitrobenzopyran.; Figure S3: (a) FT-IR spectra of the copolymer P(MAn-*alt*-OD), photochromic agent (***SP***) and P(MAn-*alt*-OD)-***SP***. (b) FT-IR characterization of the copolymers P(MAn-*alt*-2MB) and P(MAn-*alt*-2MB)-***SP***.
