# Supplementary material for: Design, Characterization, and Preparation of New Smart Photoactive Polymers and Their Capacity for Photodynamic Antimicrobial Action in Organic Film
Source: Polymers (Basel). 2025 May 3;17(9):1247. doi: 10.3390/polym17091247 (PMC12073281; doi:10.3390/polym17091247)
Supplement: Supplementary file 1 [file polymers-17-01247-s001.zip › polymers-3582374-supplementary.pdf]

Supplementary Material

Figure S1

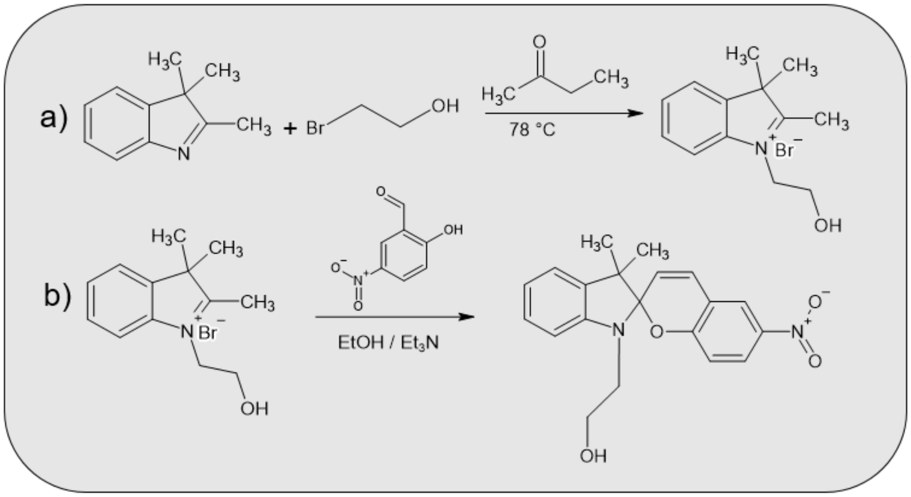

Figure **S1**. a) Synthesis of 1- (2-hydroxyethyl) -2,3,3-trimethylindolenine bromide and b) 1-(2-hydroxyethyl) -3,3-dimethylindoline-6-nitrobenzopyran.

Figure S2

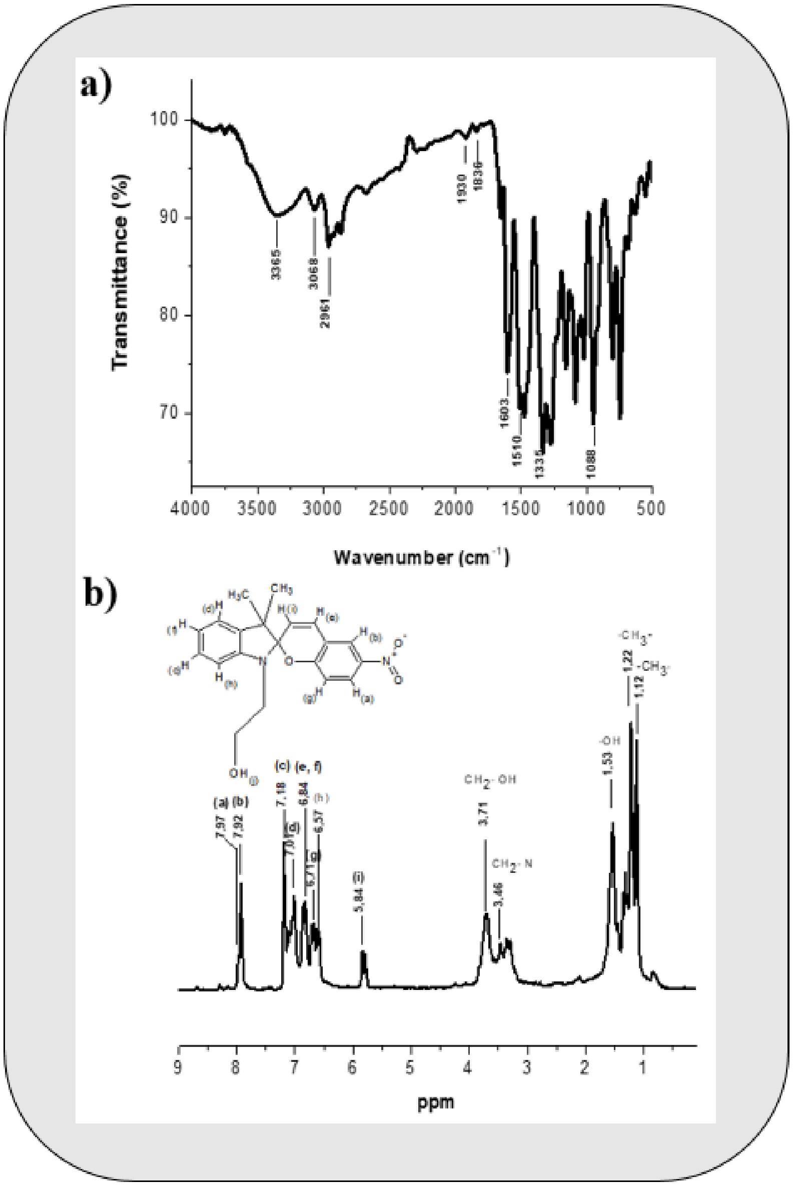

Figure **S2** a) FT-IR of 1- (2-hydroxyethyl) -3,3-dimethylindoline-6-nitrobenzopyran, b) ^{1}H-NMR spectrum of 1- (2-hydroxyethyl) -3,3-dimethylindoline-6-nitrobenzopyran.

Figure S3

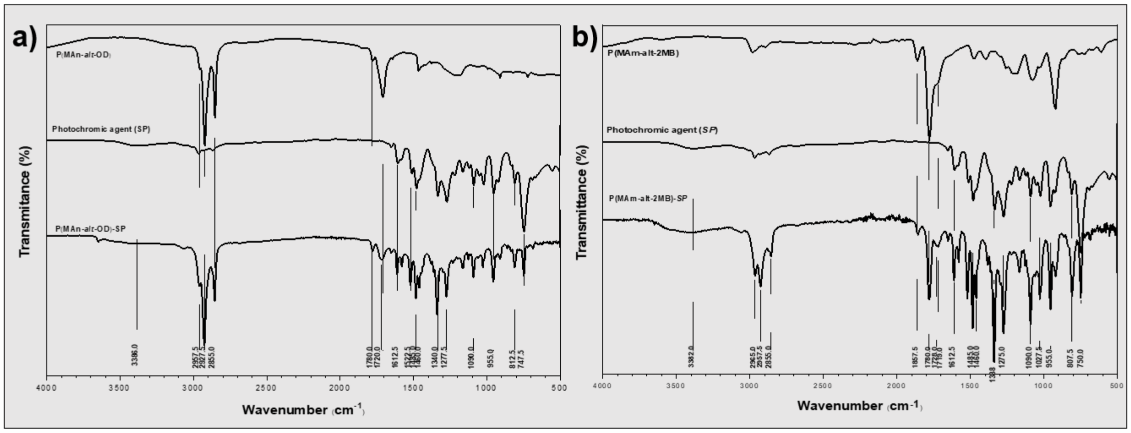

Figure S3: a) FT-IR spectra of the copolymer P(MAn-*alt*-OD), photochromic agent ***(SP)*** and P(MAn-*alt*-OD)-***SP***. b) FT-IR characterization of the copolymers P(MAn-*alt*-2MB) and P(MAn-*alt*-2MB)-*SP*.
